# The bacterial chaperone CsgC inhibits functional amyloid CsgA formation by promoting the intrinsically disordered pre-nuclear state

**DOI:** 10.1016/j.jbc.2025.110217

**Published:** 2025-05-08

**Authors:** Anthony Balistreri, Divya Kolli, Sanduni Wasana Jayaweera, Daniel Lundahl, Yilin Han, Lily Kalcec, Emily Goetzler, Rachel Alessio, Brandon Ruotolo, Anders Olofsson, Matthew R. Chapman

**Affiliations:** 1 Department of Molecular, Cellular and Developmental Biology, University of Michigan, Ann Arbor, Michigan, USA; 2 Department of Medical Biochemistry and Biophysics, Umeå University, Umeå, Sweden; 3 Department of Chemistry, University of Michigan, Ann Arbor, Michigan, USA

**Keywords:** amyloid, chaperone, inhibition mechanism, mass spectrometry, surface plasmon resonance

## Abstract

*Escherichia coli* assembles a functional amyloid called curli during biofilm formation. The major curlin subunit is the CsgA protein, which adopts a beta-sheet-rich fold upon fibrillization. The chaperone protein CsgC inhibits CsgA amyloid formation. CsgA undergoes a 3-stage aggregation process: an initial lag phase where beta-rich nuclei form, an exponential elongation phase, and a plateau phase. It is currently not known whether CsgC inhibits amyloid formation by inhibiting the formation of a pre-fibril nucleus or whether CsgC inhibits a later stage of amyloid formation by blocking monomer addition. Here, CsgC homologs from *Citrobacter youngae*, *Cedecea davisae*, and *Hafnia alvei* were purified and characterized for their ability to interrogate CsgA amyloid formation. Each of the CsgC homologs prolonged the lag phase of *E. coli* CsgA amyloid formation, similar to *E. coli* CsgC. Additionally, we found *E. coli* CsgC interacted transiently and weakly with a monomeric, pre-nucleus species of CsgA, which delayed amyloid formation. A transient CsgC-CsgA heterodimer was observed using ion mobility-mass spectrometry. When CsgC was added to actively polymerizing CsgA, exponential growth commonly associated with nucleation-dependent amyloid formation was lost. Adding preformed CsgA seeds did not rescue exponential growth, indicating that CsgC also has inhibitory activity during fibril elongation. Indeed, CsgC interacted strongly with CsgA fibers, suggesting that the interaction between CsgC and CsgA fibers can slow new fiber growth. CsgC displays unique inhibitory activity at multiple stages of amyloid formation and acts as an energy-independent chaperone that transiently interacts with prefibrillar CsgA and an amyloid fiber.

Amyloids are fibril protein aggregates that are characterized by their distinct morphology, stability, and tinctorial properties (1). Abnormal accumulation of amyloids can lead to a wide range of pathologies, including diseases such as Parkinson’s Disease, Alzheimer’s Disease, and type II Diabetes (2). The proteins that comprise amyloid fibers are often intrinsically disordered, or have intrinsically disordered regions, which adopt a repetitive cross-β strand structure upon fibrilization (2). Amyloid intermediates are cytotoxic and have been shown to be more toxic than mature amyloid fibers (3, 4). While inhibiting disease-associated amyloid formation remains a significant focus of research, a growing body of work is focused on utilizing functional amyloids as a model for amyloid formation and regulation.

Functional amyloids are a class of amyloids that provide a useful function for the cell, the list of which continues to grow (5, 6, 7). The most widely studied functional amyloid is curli, the main protein component of the *Escherichia coli* biofilm matrix (6). Curli fibrils surround cells in the biofilm and protect from desiccation, predation, and viral infection (6). The *E. coli csg* operons contain seven curli-specific genes that function as the producer and regulator for curli formation, with each protein playing a significant role (8). The main amyloid protein, CsgA, is translocated into the periplasm by the SecYEG pore (6). Periplasmic CsgA is then shepherded to the nonameric CsgG outer membrane pore by CsgE, a periplasmic chaperone (9). Two auxiliary proteins, CsgB and CsgF, are also exported through the CsgG pore and act as a nucleator and anchor, respectively, to allow CsgA amyloid fibers to form and attach to the cell’s outer surface (10, 11). Finally, the operon contains a second periplasmic protein, CsgC, which functions as a potent inhibitor of CsgA and CsgB amyloid formation (12).

CsgC is a 110-amino-acid protein with an immunoglobulin-like β-sandwich structure and proposed chaperone activity (13). *E. coli* CsgC is an efficient sub-stoichiometric inhibitor of amyloid formation against its native client *E. coli* CsgA (12). CsgC can also inhibit amyloid formation by *E. coli* CsgA homologs, the *Pseudomonas* amyloid FapC, and the human disease-associated amyloid alpha-synuclein (12). Little is known about the CsgC–client protein interaction, except (i) the interaction is guided by electrostatic interactions (14) and (ii) there is a weak client sequence determinant (12). Many details about CsgC-mediated amyloid inhibition remain poorly understood. CsgC is a simple protein with no known predicted active sites, domains, or significant amino acids. CsgC requires no hydrolysable substrate, cofactor, or cellular energy to perform its activity. CsgC does not take up a substrate or produce a quantifiable product; the only evidence of CsgC activity is the inhibition of amyloid formation. Therefore, CsgC represents a potentially novel class of anti-amyloid chaperone proteins that do not utilize an energy source and do not maintain a stable interaction with the client protein.

Amyloid formation occurs in a series of steps, during which soluble, disordered monomers join an insoluble, ordered amyloid fiber. In a solution containing amyloid-competent monomeric proteins, a low-abundance species arises from the pool of disordered monomers, which acts as a nucleation site for fiber formation (15). This nucleation event represents the rate-limiting step for amyloid formation. Disordered monomers add on to these “nuclei” and begin the progressive and thermodynamically favorable process of forming amyloid fibers (15). Fiber formation marks the transition of amyloid proteins from the soluble state to the insoluble, and in the case of curli fibrils, a transition from an intrinsically disordered monomeric conformation to a stable and ordered β-sheet-rich aggregate.

Previous work suggested that CsgC inhibits CsgA amyloid formation *via* electrostatic interactions (14), however, it is unclear what stage(s) of amyloid formation are inhibited by CsgC and what conformer of CsgA that CsgC binds to (14, 16, 17, 18). Here, we used several biochemical and biophysical techniques to provide direct evidence that CsgC can inhibit CsgA amyloid formation through two different mechanisms. We tested CsgC homologs from four Gammaproteobacteria and found that they inhibit *E. coli* CsgA amyloid formation kinetics. CsgC transiently binds a sub-population of monomeric CsgA on-pathway to form amyloid, which prolongs the formation of the aggregation-prone β-sheet rich fold state characteristic of amyloid proteins. CsgC also strongly binds CsgA fibrils and blocks further fibril elongation. We propose that these chaperone mechanisms are in accordance with the *in vivo* function of CsgC: preventing the formation of cytotoxic amyloid aggregates in the bacterial periplasm and acting as an amyloid fibril formation inhibitor.

## Experimental procedures

### Bacterial growth

All overnight cultures were grown in LB supplemented with 100 μg/ml ampicillin and/or 50 μg/ml kanamycin at 37 °C with shaking at 220 rpm. When necessary, LB plates were supplemented with ampicillin 100 μg/ml or kanamycin 50 μg/ml.

### Strains and plasmids

The full list of strains, plasmids, and primers can be found in the Supporting Information. Gibson Assembly was used to construct plasmids using the NEBuilder HiFi DNA Assembly Cloning Kit (Cat. No. E5520S). Primers were designed using the NEBuilder web tool (https://nebuilder.neb.com) and purchased from IDT (https://www.idtdna.com). Mutagenized plasmids were constructed in the MC1061 cell background. Correct mutations were confirmed using Sanger sequencing provided by Eurofins (https://www.eurofins.com/genomic-services/our-services/custom-dna-sequencing/). Plasmids were extracted from transformants using Promega PureYield Plasmid Miniprep System (Cat No. PRA1223). Miniprepped plasmids were transformed into an expression strain (NEB3016) for purification.

### Protein purification

CsgA was purified as described previously (19). CsgC and its variants were purified as described previously (20). Size exclusion chromatography was performed for CsgC purification as previously described (21). Briefly, Ni-NTA affinity chromatography elution fractions of CsgC were pooled, concentrated, and passed through a 0.22-μm filter. The sample underwent gel filtration using a Superdex 75 10/300 Gl column (Cat No.45–002–903) attached to an Äkta pure protein purification system. Elution fractions with the UV detector (A220) signal were collected. Samples corresponding to elution peaks were analyzed using SDS-PAGE. Final protein concentration of the elution fractions containing CsgC was determined using a Pierce BCA Protein Assay Kit (Cat No. 23225).

### Denaturing gel electrophoresis and dot blot

Purified protein samples were diluted in 4X SDS loading buffer and run on a 15% SDS PAGE gel with 10 μl loaded into each well (21). The gels were stained with Coomassie blue dye to visualize protein bands. Dot blots were performed by spotting a nitrocellulose membrane with 2 μl of protein and allowing the spots to fully dry. Blots were blocked for 1 hour in a mixture of TBST and skim milk. Blocked blots were then washed three times with TBST and probed with a primary antibody solution against CsgA (1:12,000) (21) or CsgC (1:4000) (16). Primary antibody was removed from blots, which were then washed three times with TBST prior to the addition of a secondary antibody solution. Secondary antibodies against rabbit IgG and conjugated with IRDye 800CW (Cat No. NC9401842) were used to image the blots in a Licor Odyssey FC.

### Surface plasmon resonance

Surface plasmon resonance (SPR) experiments were performed on a BIAcore 3000 (GE Healthcare) using a CM5 chip for the immobilization of sonicated CsgA fibrils and gel-filtered CsgA monomers. The CsgA monomer used was a variant protein (CsgA_CC_) with two cysteine residues that lock CsgA into a non-aggregative conformation unless incubated under reducing conditions (22). Immobilization was carried out following protocols from previous fibrillar SPR studies (23), with CsgA fibrils immobilized at 1300 RU and monomers at 500 RU. After immobilization, the flow cells were probed with gel-filtered CsgC at concentrations ranging from 4 μM to 0.25 μM (two-fold dilutions). CsgC was injected for 5 min over the CsgA fibrils and 7.5 min over the CsgA monomers. The experiment was conducted in phosphate buffer (pH 7.5) containing 0.05% Tween, at a temperature of 25 °C. Prior to immobilization, CsgA fibrils were sonicated for 1 min in a water bath.

### Preparation of CsgC-modified or Tris-quenched NHS columns

CsgC was purified as described previously (20). NHS-activated agarose beads were buffer exchanged into 50 mM potassium phosphate buffer (pH = 7.4) and reacted with purified CsgC (1 mg CsgC/ml NHS-activated agarose beads, Cat. No. 26200) overnight at 4 °C with gentle rocking. The reacted resin was then washed with 50 mM potassium phosphate buffer, pH = 7.4, to remove any unbound CsgC and cleaved NHS-groups. Reaction progress was tracked by 260 nm and 220/280 nm absorbance values to measure the increase in cleaved NHS-groups and decrease in free CsgC protein, respectively. SDS-PAGE and Pierce BCA Protein Assay Kit (Cat No. 23225) were used to ensure complete binding of CsgC to the resin. Unreacted NHS groups still bound to agarose beads were cleaved by the addition of a quenching buffer (1M Tris HCl, pH 7.5) and incubated for 30 min at room temperature with gentle rocking. The fully reacted resin was buffer exchanged into 50 mM potassium phosphate buffer, pH = 7.4, to remove quenching buffer. 260 nm and 220/280 nm absorbance values were measured to ensure complete quenching of unreacted NHS-linked agarose beads. Modified NHS columns were stored at 4 °C until use. Individual columns were only used once for each assay.

### Sample preparation for ion mobility-mass spectrometry (IM-MS)

Purified CsgA and CsgC were buffer exchanged into 20 mM ammonium acetate (pH 7.4) using Thermo Scientific Zeba Spin Desalting Columns 7k MWCO (Cat No. 89892). The protein concentration after buffer exchange was assayed using the Thermo Scientific Pierce Rapid Gold BCA Protein Assay Kit. CsgA and CsgC were each diluted to 20 μM with 20 mM ammonium acetate, mixed at a 1:1 ratio. The mixture was incubated at 37 °C for 23 h. Time points were taken at 0 h, 3 h, 6 h, and 23 h.

### IM-MS

IM-MS data were collected on a quadrupole ion-mobility time-of-flight (TOF) mass spectrometer (Synapt G2 HDMS, Waters) with a nano-electrospray ionization (nESI) source. The source was operated at positive mode with the nESI voltage set at 1.0 to 1.2 kV, the sampling cone was set to 15 V, and the bias was set to 42 V. The source temperature was set to 20 °C. The traveling–wave ion mobility separator operated at a pressure of approximately 3.4 mbar with wave height and wave velocity set at 30V and 500 m/s, respectively. The m/z window was set from 100 to 8000 m/z with a TOF pressure of 1.5e-6 mbar. Mass spectra were analyzed using MassLynx 4.1 and Driftscope 2.0 software (Waters). CCS (Ω) measurements were externally calibrated using a database of known values in helium. We reported the standard deviations from replicate measurements of CCS and an additional ±3% to incorporate the errors involved in the calibration process.

### Thioflavin T Binding assay

Assays were performed as previously described (19). Briefly, freshly purified CsgA was diluted with phosphate buffer to 20 μM or 10 μM and combined with an excess of the amyloid-specific dye thioflavin-T (ThT) (Cat No. AC211760250). Amyloid formation was monitored by measuring an increase in ThT fluorescence at 495 nm (450 nm excitation). Assays were performed in triplicate at microscale within 96-well plates and measured with Infinite Pro M200 or Infinite Nano^+^ F200 Tecan plate readers. CsgC proteins were purified, diluted with phosphate buffer, and added to specified assays in the reported stoichiometric ratio. Fiber-stimulating “seeds” were produced by purifying WT CsgA and allowing fiber formation for at least 72 h. The fibers were sonicated directly for 10 s on and off two times before adding to a specified assay. Time to half-maximum (T_1/2_) is calculated by the time at which the Thioflavin-T fluorescence reaches half of the maximum Thioflavin-T fluorescence. Duration of lag phase (T_lag_) was calculated as described by Arosio *et al.* (24). Briefly, T_lag_ is calculated by T_1/2_ subtracted by half of the maximum of the first derivative of the sigmoidal ThT fluorescence.

### Circular dichroism

CsgA was diluted with phosphate buffer to a final concentration of 15 μM. Secondary structure was measured at room temperature every 60 min (in triplicate) with the JASCO-1500 using default parameters. Measurements were averaged, the baseline signal of phosphate buffer was subtracted, and the curve was smoothed using a Savitzky-Golay filter. Final curves were normalized by concentration and path length.

## Results

### *E. coli* CsgC and three CsgC homologs affect multiple stages in the CsgA amyloid formation pathway

**Figure 1:**
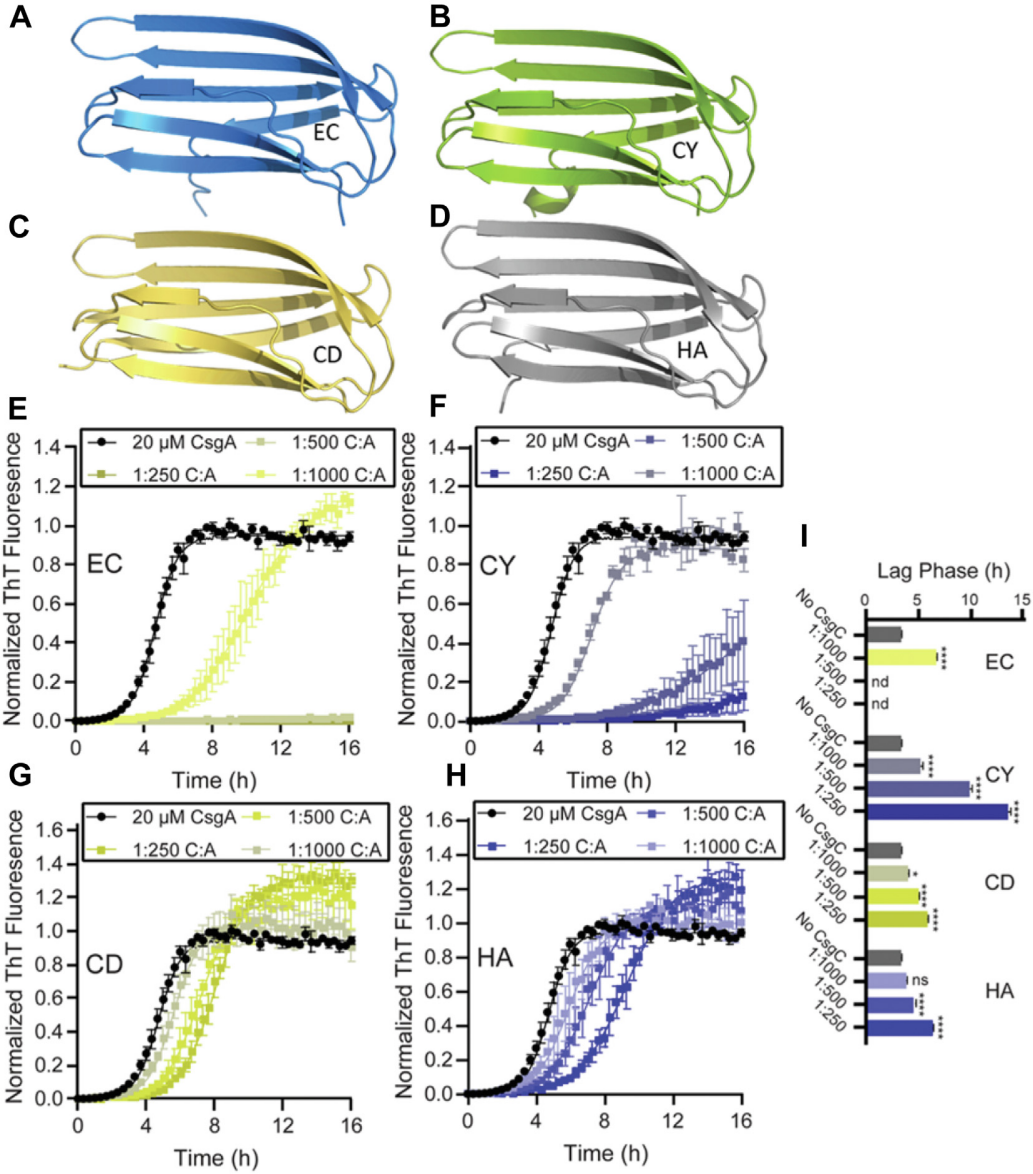
**Comparing inhibition of *E. coli* CsgA amyloid formation by EC CsgC and its homologs.***A*–*D*, AlphaFold 2.0 predicted structures for CsgC EC, CY, CD, and HA. *E*–*H*, four different CsgC homologs were purified and tested for their ability to inhibit CsgA *in vitro*. In all cases, 20 μM CsgA was freshly purified and mixed in the stated stoichiometric ratio with CsgC homologs. The data points shown represent the average of triplicate experiments, the error bars show the SEM, and the curves were fit using the sigmoidal logistic function described in (24). *I*, the calculated lag phase for each homolog according to the fit curves. The error bars represent the SEM for each value. “nd” was used to denote values that were not defined by the equation used. Significance was attributed using a one-way ANOVA analysis comparing all values to the uninhibited CsgA condition; ∗*p* < 0.05, ∗∗∗∗*p* < 0.00005.

CsgC was first identified in *E. coli* (EC), but *csg* operons from other Gammaproteobacteria contain CsgC homologs (25). *Citrobacter youngae* (CY), *Cedecea davisae* (CD), and *Hafnia alvei* (HA) are three Gammaproteobacteria that have *csg* operons containing homologs to EC CsgC. These four CsgC homologs have similar structures, as predicted by Alphafold 2.0 (26); however, they share a range of sequence identity with EC CsgC: CY (69%), CD (51%), and HA (35%) (Figs. 1, *A*–*D* and S1). EC CsgC and each homolog was tested for inhibitory activity against *E. coli* CsgA using ThT-binding assays. EC CsgC was the most efficient inhibitor of *E. coli* CsgA amyloid formation, with full inhibition at a 1:500 stoichiometric ratio (40 nM) (Fig. 1*E*). CY CsgC, CD CsgC, and HA CsgC all inhibited CsgA aggregation to a lesser extent (Fig. 1, *F*–*H*). All the ThT assay results were fit with a sigmoidal function curve. We derived the duration of the lag phase for each curve as a proxy for the nucleation rate of CsgA polymerization (24). The lag phase of *E. coli* CsgA was significantly prolonged upon addition of CsgC homologs in nine of the 10 assays where a lag phase could be calculated (Fig. 1*I*). The lag phase was not determined in two cases because the ThT signal never increased within the time frame of the experiment (Fig. 1*I*). The only condition where the addition of CsgC did not affect the lag phase was with the addition of the lowest ratio (1:1000) of HA CsgC homolog, which has the lowest sequence identity to EC CsgC (Fig. 1, *H* and *I*).

Amylofit was used for additional analysis of the ThT curves produced in Figure 1. Amylofit is a global fitting software that fits ThT binding assay curves, calculates estimated values for reaction orders, rate constants, and more to describe amyloid aggregation kinetics (27). Meisl and coworkers suggest that Amylofit can be used to determine the molecular species or stage along an aggregation pathway that is affected by an inhibitor (27). The rate expressions used to fit ThT curves describe the formation of CsgA amyloid fibers starting with unfolded CsgA monomers:

(1) $${\text{CsgA}}_{Disordered}\overset{\overset{k_{n}}{\to }}{\leftarrow }{\text{CsgA}}_{Nucleus}$$

(2) $${\text{CsgA}}_{Nucleus}+{\text{CsgA}}_{Monomer}\overset{\overset{k_{+}}{\to }}{\leftarrow }{\text{CsgA}}_{Fiber}$$

A primary nucleus forms in solution from an unknown number of folded CsgA monomers (k_n_, eq. 1). After a critical concentration of nuclei are present, CsgA monomers use nuclei as folding templates, sparking rapid fiber elongation (k_+_, eq. 2). For some amyloid proteins, the presence of amyloid fibers in solution stimulates new fiber growth (this could be described by rate constant k_2_).

We inputted the same data displayed in Figure 1, *E*–*H* into Amylofit and normalized each curve to reflect the final aggregate amount in the uninhibited CsgA condition. The “secondary nucleation dominated” model was used to fit the ThT curves because, under these aggregation conditions, this model provided the best fit. First, all rate constant parameters (k_n_, k_2_, and k_+_) were allowed to be fit individually, providing the best-fit curves for each condition (Figs. S2*A*, S3*A*, S4*A* and S5*A*). Next, all parameters except one were set to a global constant using the values derived from CsgA fibril formation in the absence of inhibitor. This was repeated for all rate constant parameters. Using this method, each rate constant parameter was set to individually fit each inhibited condition (Figs. S2, *B*–*D*, S3, *B*–*D*, S4, *B*–*D*, and S5, *B*–*D*). This method illustrated the degree to which each rate constant can explain changes in the ThT curve upon addition of increasing concentrations of inhibitor (27). Best-fit curves were created for each condition and the mean squared residual error (MRE) results from each condition provided a global metric for goodness of fit for all curves (27). Low MRE values are an indication that the fitted rate constant is affected by the addition of the inhibitor. MRE values for each individually fit rate constant were generally close to the universal best fit MRE value (Figs. S2*E*, S3, *A* and *E*, S4*E*, and S5*E*); however, all homologs had the lowest MRE value associated with either the rate of secondary nucleation (k_2_) or elongation (k_+_) rather than primary nucleation (k_n_). This suggests that CsgC could be affecting CsgA aggregation at the fibril growth level. Taken together, Figures 1 and S2–S5 indicated that CsgC may affect CsgA amyloid formation at multiple stages. Hereafter, any reference to CsgC will refer to EC CsgC.

### CsgC partially inhibits CsgA amyloid formation during the elongation phase

CsgA amyloid formation follows a primary nucleation-dependent polymerization mechanism that has three distinct phases: an initial lag phase, an elongation phase, and a stationary phase. The elongation phase is marked by fibril elongation directed by soluble protein monomer addition onto fiber ends (28, 29). During the elongation phase, there are intrinsically disordered CsgA monomers still in solution capable of either primary nucleation or addition onto a fiber (30).

**Figure 2:**
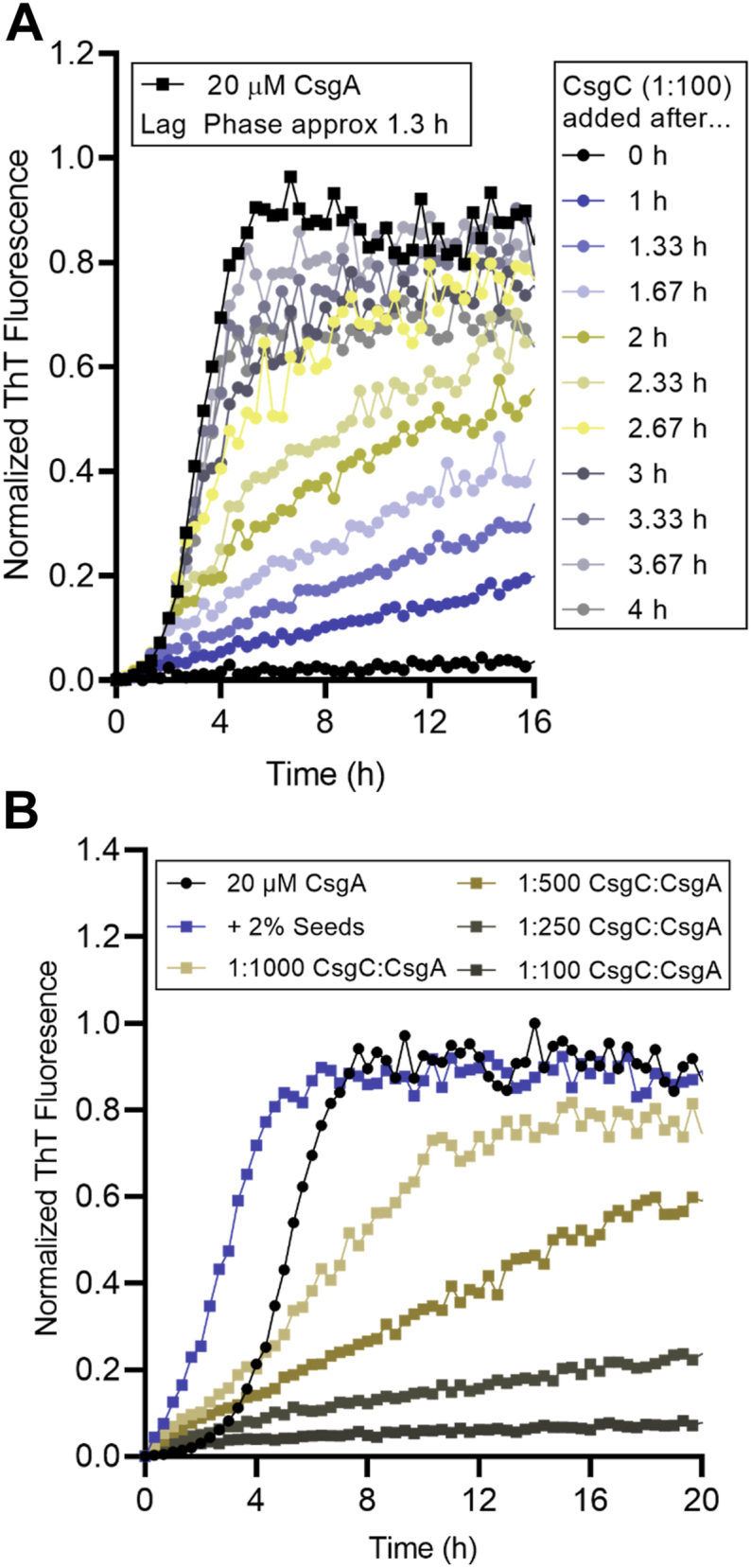
**Addition of CsgC to actively polymerizing CsgA.***A*, 200 nM (1:100) CsgC was added to actively polymerizing CsgA at the times indicated and amyloid aggregation was monitored in a ThT binding assay. *B*, in a similar assay, CsgC was added to a 2% (400 nM) seeded reaction of CsgA at various ratios as indicated in the key above (All olive conditions also contain 2% seeds).

CsgC was added at different time points to actively polymerize CsgA to determine how CsgC inhibitory activity changes throughout amyloid formation. First, 200 nM CsgC was added to a solution of 20 μM CsgA (1:100 CsgC: CsgA molar ratio) at different time points after the start of CsgA amyloid formation (Fig. 2*A*). When CsgC was added at time 0 h, the ThT signal remained very low compared to CsgA samples without CsgC, indicating nearly total inhibition. When CsgC was added to CsgA after 1 h, the ThT signal increased linearly (Fig. 2*A*). When CsgC was added after the lag phase had ended, the result was a linear increase in ThT signal followed by a gradual decrease reaching a plateau in signal (Fig. 2*A*). However, it is not clear whether the inhibition of actively polymerizing fibrils is due to the prevention of new nuclei formation or the prevention of fibril elongation.

CsgC was added to CsgA that had been seeded by the addition of preformed CsgA fibers to determine if CsgC inhibitory activity is based on preventing new nuclei formation or fibril elongation (Fig. 2*B*). During a seeded reaction, CsgA is presented with preformed fibers which act as a template for amyloid formation, effectively diminishing the need for primary nucleation and the lag phase entirely. CsgA was purified and allowed to form fibrils. These fibrils were then sonicated to form “seeds” and added at a concentration of 400 nM (2%) to a solution of 20 μM freshly purified CsgA (Fig. 2*B*, dark blue squares compared to black circles) (31). When CsgC was added to the seeded CsgA reaction, the result was a linear increase in ThT signal (Fig. 2*B*), indicating that CsgC may have an inhibitory activity independent of nucleus formation.

### CsgC binds to CsgA fibrils but not to CsgA monomers

**Figure 3:**
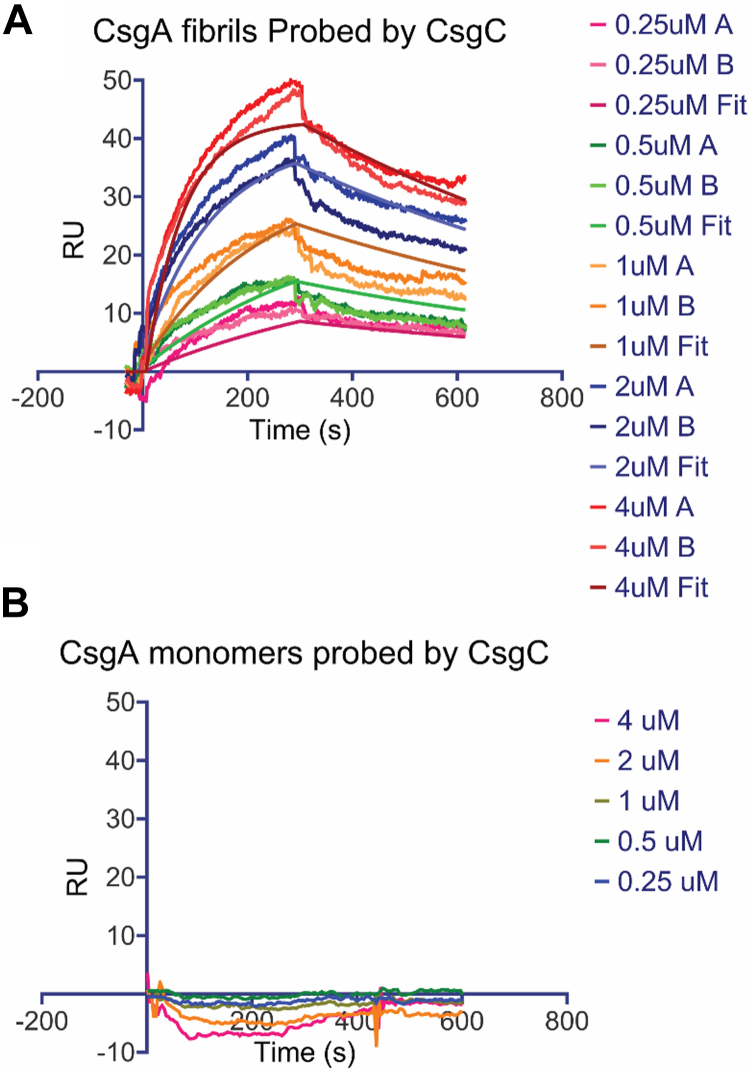
**Sensograms of CsgA fibrils and monomers probed by CsgC.***A*, sensogram data of CsgA fibrils and CsgC, showing an equilibrium dissociation constant (K_D_) of 360 ± 10 nM. The concentrations labeled “*A*” represent the first injection, while “*B*” denotes the secondary injection. The fitted curve represents the curve fitting performed using Scrubber2. *B*, sensogram data of CsgA monomers and CsgC across various tested concentrations. Residual standard deviation = 3194 RU.

Surface plasmon resonance was used to determine the binding kinetics between CsgC and CsgA monomers or CsgA fibrils. Surface plasmon resonance can interrogate interactions between proteins and calculate a K_D_ (32). Either CsgA fibrils (Fig. 3*A*) or CsgA monomers (Fig. 3*B*) were bound to a CM5 chip using standard amino-coupling reaction. A double cysteine variant of CsgA (CsgA_CC_) that retains an intrinsically disordered structure under oxidizing conditions was used to prevent bound monomeric CsgA from aggregating during the course of the SPR experiment (22). Solutions containing freshly purified monomeric CsgC were flowed over the sensors.

The sensogram data with curve fits is shown in Figure 3. The residuals indicated a generally good fit, with only minor deviations observed. Notably, concentrations below 250 nM were excluded from the kinetic analysis due to detection limitations, as the responses at these lower concentrations were too weak to be reliably measured by SPR. Sensogram data indicated that monomeric CsgA had no measurable binding to monomeric CsgC (Fig. 3*B*). However, monomeric CsgC interacted with CsgA fibrils with a dissociation rate of k_d_ = (1,18 ± 0.05) × 10^−3^ s^−1^ and an equilibrium dissociation constant of K_D_ = 360 ± 10 nM (Fig. 3*A*).

### Transient interaction between monomeric CsgC and non-fibrillar CsgA is sufficient to delay amyloid formation

Previous studies have shown that CsgA incubated with CsgC does not readily form higher-order fibrillar aggregates (14, 16). These data, in conjunction with data in Figure 1 and S2–S5, indicated that CsgC can also inhibit the initial conversion of monomeric CsgA into an oligomeric amyloid. Due to the undetectable binding between monomeric CsgC and CsgA from Figure 3, we utilized a modified pull-down assay to determine whether CsgC interacts transiently with prefibrillar CsgA.

**Figure 4:**
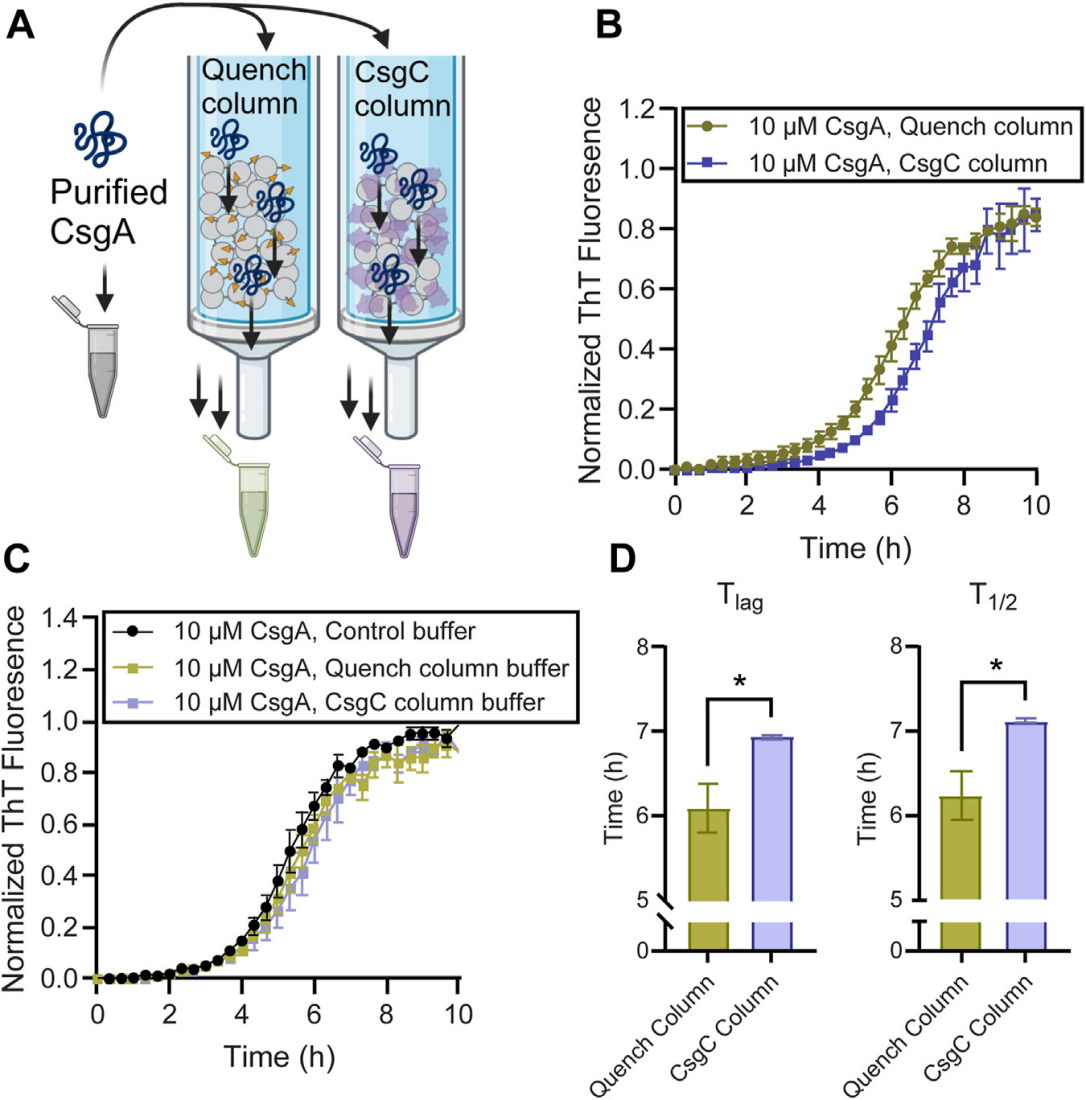
**Passing monomeric CsgA by immobilized CsgC delayed the formation of ThT-positive aggregates.***A*, affinity-purified CsgA was passed through a column of NHS agarose resin that was either amino-coupled to CsgC or Tris base to quench the free NHS binding spots. *B*, eluent CsgA was then diluted to 10 μM in phosphate buffer for a ThT binding assay to observe amyloid aggregation kinetics. *C*, quenched and CsgC resin was incubated in phosphate buffer for 24 h. The incubated phosphate buffers were used to dilute freshly purified CsgA to 10 μM and added to a ThT binding assay. *D*, a measurement of the lag phase (T_lag_) and time to half maximum fluorescence (T_1/2_) was calculated using a sigmoidal curve function. *Error bars* represent the standard error of the mean across three technical replicates. Significance was attributed using a student’s *t* test analysis comparing the two conditions; ∗*p* < 0.05.

*N*-hydroxy-succinimide (NHS) resin beads were coupled to CsgC protein for the modified pull-down assay. Another batch of *N*-hydroxy-succinimide (NHS) resin beads was quenched with Tris to use as a control. Immediately after Ni-NTA and SEC purification, a CsgA sample was split into equal parts. One aliquot of the purified CsgA was left on ice, while the other aliquot was passed through a column containing either the CsgC-linked beads or the Tris quenched beads (Fig. 4*A*). CsgA eluent from the column was then diluted into the wells of a ThT binding assay to monitor amyloid formation. The CsgA from the Tris-quenched resin condition began aggregating at 2 h, consistent with CsgA that had been left on ice (Fig. S6*A*). Interestingly, the CsgA that passed by the CsgC-linked column had a diminished propensity to form amyloid, displaying an approximately 77 min increase in ThT lag phase and a 75 min increase in the time it takes to reach half maximum ThT fluorescence compared to CsgA that had been left on ice (Fig. S6*B*). CsgA eluted from the CsgC-linked column displayed a 51 min increase in the lag time when directly compared to CsgA eluted from the Tris-quenched column and a 52 min increase in the time it takes to reach half maximum ThT fluorescence, displaying that transient interaction between CsgA and CsgC is sufficient for delayed amyloid formation (Fig. 4, *B* and *D*). Buffer was eluted from the Tris-quenched column and the CsgC-linked column and added into a ThT binding assay with freshly purified CsgA to ensure that no CsgC, NHS, or Tris eluted from the resin that could affect CsgA aggregation (Fig. 4*C*).

CsgA protein concentration was quantified prior to, and following, elution from each resin to determine how much CsgA was retained on the CsgC-linked beads. Little to no difference was observed between the eluent concentration of CsgA eluted from the control Tris-linked resin beads (31.2 μM) and the eluent concentration of CsgA eluted from the CsgC-linked resin beads (25.7 μM). To determine if the small difference in concentration was due to CsgA retention on the CsgC-linked resin column, the CsgC-linked resin and Tris-linked resin was treated with hexafluoroisopropanol (HFIP) to digest CsgA aggregates left on the resin beads. Samples were then analyzed by a dot blot and SDS-PAGE to determine if any CsgA is retained on the beads following elution. Both the SDS-PAGE gel and the dot blot indicated that no detectable CsgA was retained on the resin following elution (Fig. S7). This further supports that CsgC transiently interacts with CsgA and that this interaction is sufficient to observe the extended lag phase in Figure 4.

Additionally, the change in CsgA secondary structure from disordered to beta-sheet rich was visualized using circular dichroism (Fig. S8). Amyloid protein folding is often assessed using CD by measuring the formation of a spectral minimum at 220 nm, indicating the formation of a beta-sheet-rich and amyloidogenic secondary structure. No significant effect of CsgC transient interaction on beta-sheet structure formation was detected by circular dichroism. With the indication of an interaction between CsgC and CsgA monomers by ThT but not by CD, we decided to look more closely at their interaction using a highly sensitive native mass spectrometry technique.

### A transient 1:1 CsgC: CsgA complex is detected

**Figure 5:**
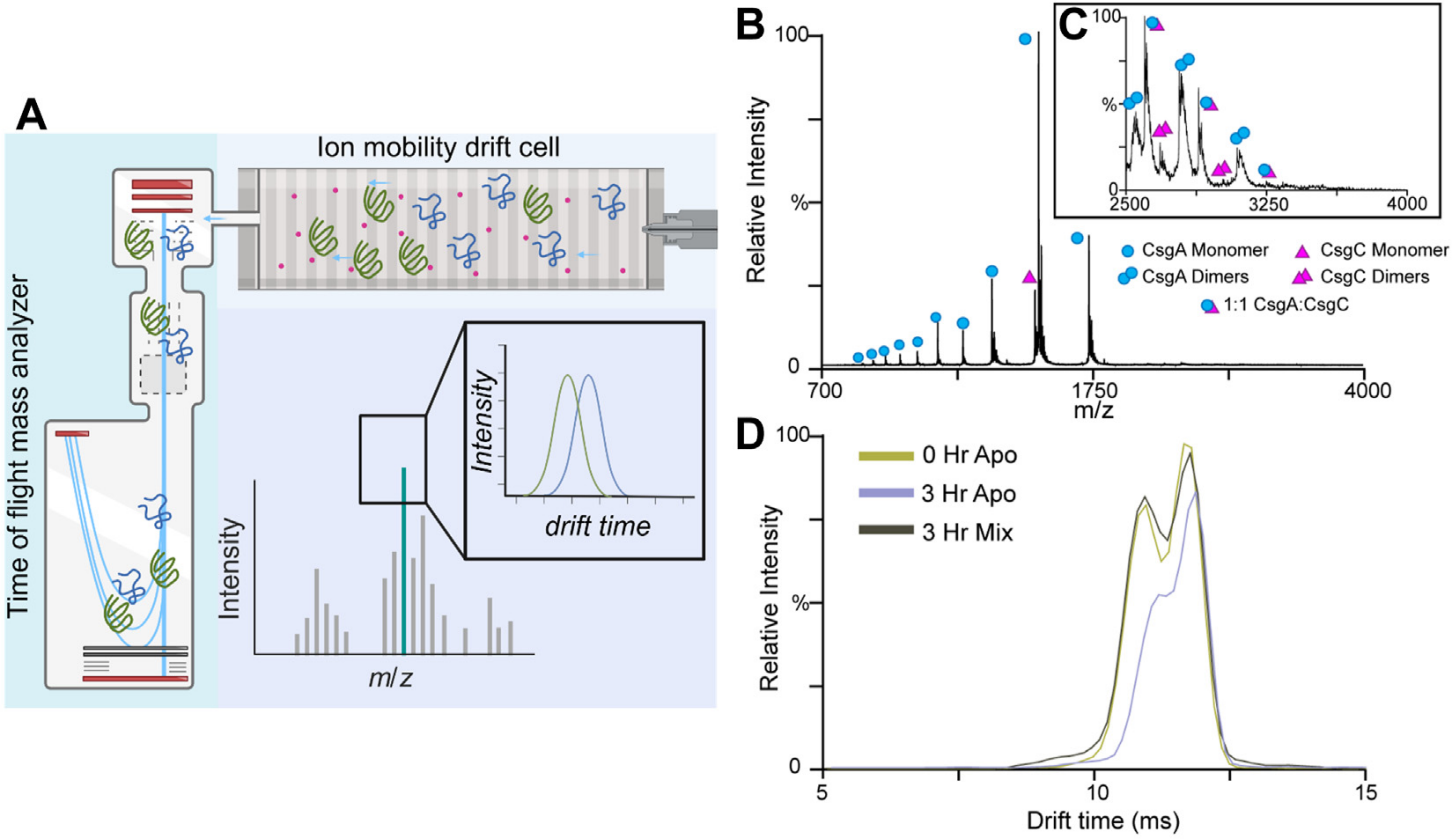
**IM-MS to detect CsgC-CsgA interaction.***A*, proteins were passed through an ion mobility drift cell that separates based on size. Next, proteins pass through a time-of-flight mass analyzer. In combination, IM-MS can provide three modes of information for a given ion: mass to charge ratio, drift time, and the intensity of species with those properties. *B*, mass spectra for CsgA incubated with CsgC in a 1:1 M ratio at 37 °C. Monomeric CsgA (*blue circles*), dimeric CsgA (*blue double circles*), monomeric CsgC (*purple triangles*), dimeric CsgC (*double purple triangles*) and 1:1 CsgA:CsgC complexes (*blue circle* and *purple triangle*) are annotated. A magnified MS spectrum (*C*) showed that the complex is seen flanked on either side by dimeric CsgA and dimeric CsgC. *D*, arrival time distribution of the 11+ monomer of CsgA in three experimental conditions: Apo CsgA in solution at 0 h (*olive trace*), apo CsgA in solution after 3 h (*light purple trace*), and CsgA in solution with CsgC after 3 h (*dark grey trace*). Traces are overlapped to show the changes in ATD after 3 h of incubation.

Native mass spectrometry (native MS) uses electrospray ionization conditions to preserve non-covalent protein-protein and protein-ligand complexes (33). The added dimension of the ion mobility (IM) spectrometry allows ions to be separated based on their size, shape, and charge (Fig. 5*A*) (34). We purified both CsgC monomers and CsgA monomers, added them together in a 1:1 mixture, and analyzed the resulting solution for any interactions between the two proteins. During our first time point, we observed monomeric and dimeric species of both proteins, including low-abundance heterodimeric complexes of CsgA and CsgC (Fig. 5, *B* and *C*). CsgA exhibited a broad charge state distribution ranging from 5+ to 13+, as typically observed for intrinsically disordered proteins (Fig. 5*B*) (35). The CsgA: CsgC mixture remained soluble throughout the 23 h time course, while apo CsgA, serving as a control sample, formed micro-scale aggregates that led to the clogging of the nESI emitters and prevented the collection of both 6 h and 23 h time point data. An analysis of collisional cross-section (CCS) values of CsgA monomers showed very little difference between time 0 and 3 h (Fig. S9*A*). Similarly, we also saw no changes in CCS for CsgC monomer ions through the 23 h time course (Fig. S9*B*).

We then analyzed the IM arrival time distributions (ATDs) to assess conformational changes within CsgA and CsgC over time. The ATD data contains information regarding both ion shape and the number of conformer families present for a single protein ion population (36). Data shown in Figure 5*D* displays three overlaid ATD profiles for CsgA 11+ monomer ions recorded after 0 and 3 h incubations. In addition, similar IM ATD data is shown for CsgA/CsgC mixtures following a 3 h incubation. All three datasets yield a bimodal ATD profile, consisting of both a more compacted (shorter IM drift times) and a more extended conformational family (longer IM drift times). A comparison of CsgA IM data collected at both 0 and 3 h time points revealed that the peak corresponding to the more compact conformation decreased significantly in intensity, resulting in an overwhelmingly more extended protein ion population. Overall, our IM ATD data suggested that the conformational ensemble of CsgA becomes more extended as protein aggregation progresses, an observation common to other amyloid proteins (37, 38). Importantly, when we compare our IM ATD data recorded for CsgA at 0 h with ATDs recorded for CsgA/CsgC mixtures following 3 h of incubation, we detected a few significant differences, indicating that the initial, intrinsically disordered state of CsgA is better preserved in the presence of CsgC. The results discussed above were consistent across multiple CsgA charge states (Fig. S9, *C* and *D*).

## Discussion

Amyloid formation can lead to a wide range of pathologies. Therefore, it is of great interest to discover and characterize molecules that can prevent pathogenic amyloid formation. Synthetic amyloid inhibitor peptides occupy a large biochemical space, from β-blocking D-amino acid peptides (39) to camelid nanobodies (40, 41). There is a short list of naturally occurring proteins which putatively inhibit functional amyloid formation. This list includes molecular chaperones and a diverse set of human and bacterial proteins which have similar 3D structures (17). However, CsgC differs from the rest of the known amyloid inhibitor proteins in one significant way: CsgC remains the only example of an amyloid inhibitor protein that exists exclusively to moderate the amyloid formation of specific proteins. CsgC is part of the CsgBAC operon; therefore, when amyloid proteins CsgA and CsgB are expressed, the CsgC protein will also be expressed. Studying CsgC provides a unique opportunity to answer questions about how cells prevent amyloid formation and utilize functional amyloids, as well as how other structurally similar amyloid inhibitors may function.

CsgC is a highly effective anti-amyloid chaperone protein (16); however, CsgC’s mechanism of action has remained unclear. Early work found that CsgC could inhibit CsgA without the need for a hydrolysable energy source and at a low sub-stoichiometric ratio (16). The key to understanding the mechanism of CsgA amyloid inhibition is the nature of the CsgC interacting partner. We have shown in previous work that CsgC retains soluble CsgA monomers in an intrinsically disordered form (16), implying that CsgC may act prior to CsgA folding. However, a monomer-monomer interaction between the two proteins was not observed. It has also been suggested that CsgC interacts with elongating CsgA fibers (18). Protein chaperones that interfere with amyloid formation have been observed to interact with a variety of binding partners and inhibit aggregation at different steps along the amyloid formation process (42). Monomeric proteins exist in solutions during all stages of amyloid formation and can either form nuclei or add onto a growing fibril (43). Interactions between an inhibitor and an amyloid-competent monomer results in a decrease in the rate of several steps along amyloid formation (42). A systematic approach was necessary to delineate between CsgC-mediated inhibition that occurs on pre-nuclear CsgA and fibrillar CsgA to better understand how CsgC works. Collectively, our data demonstrates that there is a transient interaction between CsgC and a monomeric, prefibrillar species of CsgA as well as a more stable interaction with fibrillar CsgA.

### CsgC extends CsgA lag phase

When proteins self-assemble into amyloid fibers through a nucleation-dependent process they typically display sigmoidal kinetics (24). There are three phases to polymerization beginning with the “lag phase”, a rate-limiting period of protein folding and initial oligomerization (24). After the lag phase, a critical mass of nuclei has formed, and aggregation increases exponentially as monomers quickly add onto fiber ends and new fibers continue to sprout from nuclei. CsgA is known to act mostly through a primary nucleation pathway and not through secondary nucleation where new fibers sprout from the lateral edge of an existing fiber (17, 44, 45).

CsgA requires a longer time to begin rapid amyloid formation when CsgC is present in solution. We saw lag phase extension in almost every scenario we tested, which included four CsgC homologs in a wide range of substrate:CsgC concentrations (Fig. 1). The CsgC homologs we tested share between 68% and 33% sequence identity with *E. coli* CsgC, and yet, they all inhibit *E. coli* CsgA in a similar manner. Although the CsgC homologs have modest sequence identity, they share a striking structural identity (Figs. 1 and S1). This suggests that the mechanism of CsgC inhibition may not depend heavily on sequence determinants but, rather, the unifying 3-dimensional shape of CsgC-like proteins. Moreover, there is a distinct correlation between the concentration of the CsgC proteins and how much the lag phase was extended (Fig. 1). This indicates a true dose–response relationship between CsgC proteins and the extension of CsgA lag phase, and thereby, the extent of primary nucleation.

### CsgC inhibits new nuclei formation and fibril elongation during ThT elongation phase

CsgC remains an active amyloid inhibitor during the rapid fiber formation phase of aggregation. We show two similar experiments in Figure 2 where CsgC was presented to CsgA during the elongation phase. In the first experiment, CsgC was added to CsgA at different times throughout aggregation and we monitored the effect of CsgC addition on the increase in ThT fluorescence (Fig. 2*A*). CsgA alone takes approximately 1.3 h to transition from the lag to the elongation phase (Fig. 2*A*). When CsgC is added at Time 0, we see little to no increase in ThT fluorescence throughout the time course of the experiment, consistent with total inhibition of nuclei formation (Fig. 2*A*). For all conditions tested where CsgC was added after Time 0, providing time for CsgA to begin to form primary nuclei, there was a linear increase in ThT signal until the signal eventually reached a plateau and flattened out (Fig. 2*A*). This indicates that the addition of CsgC after initial formation of CsgA nuclei yields only a partial inhibition of CsgA amyloid formation. Linear or isodesmic polymerization is consistent with a model of aggregation that is independent of nucleation and driven by fibril elongation (46). CsgC-inhibited CsgA could present an isodesmic polymerization profile because the formation of new nuclei is being hindered, while CsgA unfolded monomers can still add to fiber ends (46, 47). However, this result can also arise due to inhibition of both new nuclei formation as well as inhibition of fibril elongation.

To determine whether CsgC inhibition is solely based on inhibition of nuclei formation, a similar experiment was performed in the presence of seeds to rescue CsgA amyloid formation. Adding preformed fiber seeds to monomeric CsgA provides a template for the monomers to quickly adopt an aggregation prone state and therefore add onto a growing fiber end. Seeds act as catalysts, effectively lowering the free energy requirements to fibril formation. If CsgC acts only by inhibiting nuclei formation, the addition of seeds should replace the need for nuclei and fully rescue CsgA fibril formation. A linear increase in ThT signal can be seen when CsgC is added to a seeded CsgA aggregation reaction (Fig. 2*B*) indicating that the addition of seeds does not recapitulate un-inhibited CsgA amyloid formation. Therefore, CsgC must be acting to inhibit nuclei formation as well as fibril elongation. Additional kinetic analysis of data shown in Figure 2*B* was performed to apply a first order kinetic model to seeded CsgA reactions that included CsgC (Fig. S10). Though the de facto binding partner of CsgC could not be determined through this analysis, it nevertheless showed that CsgC is an effective substoichiometric inhibitor of seeded CsgA reactions, displaying a K_i_^−1^ of approximately 8 nM^−1^.

**Figure 6:**
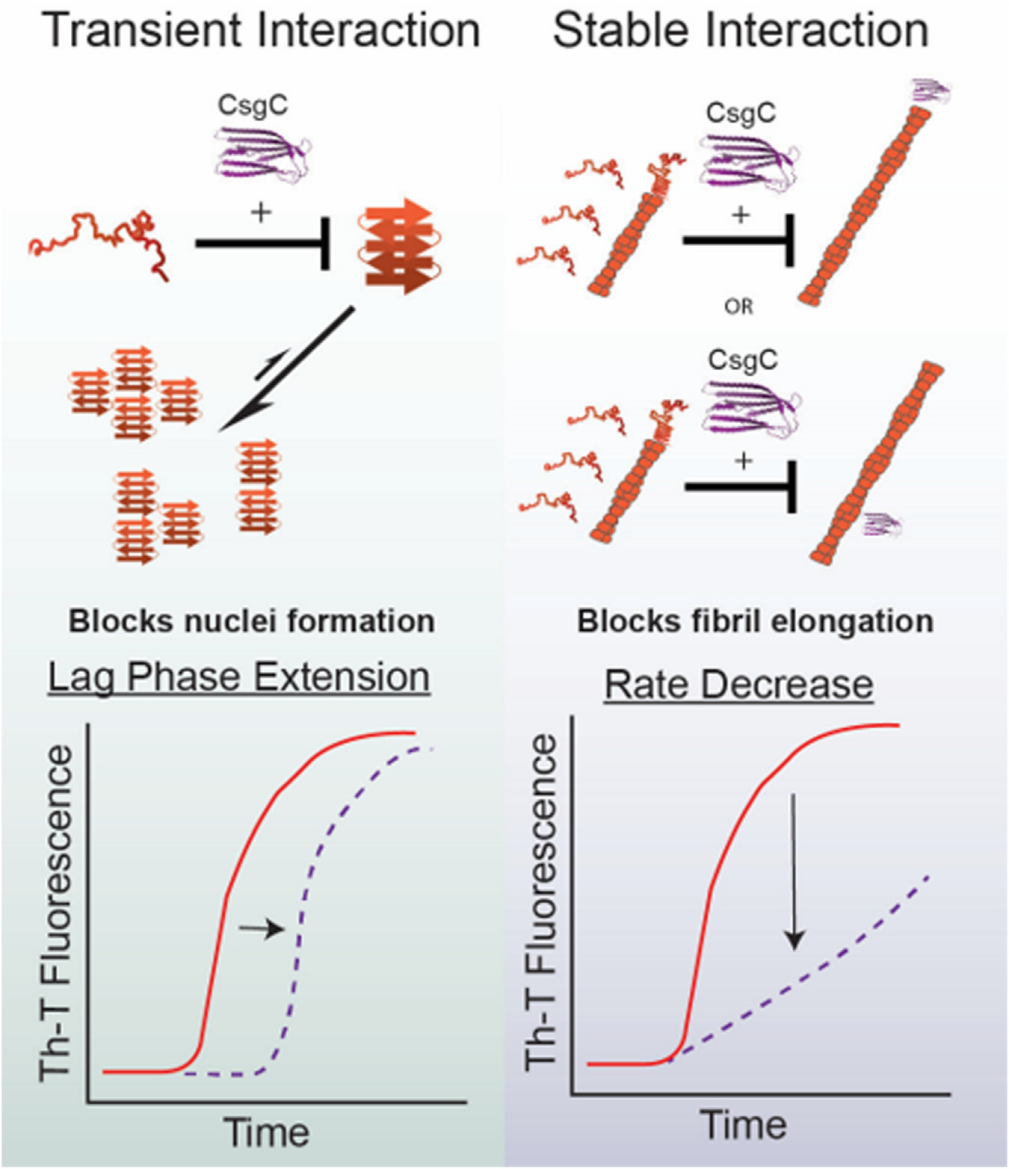
**Schematic of CsgC inhibitory mechanism.** CsgC inhibits CsgA nuclei formation leading to a prolonged lag phase. This interaction between prenuclear CsgA and CsgC is transient (*left*). CsgC can also bind stably to CsgA fibrils when added to a mixed CsgA population containing monomers, oligomers, and growing fibrils. This stable interaction prevents fibril growth by either preventing fibril elongation or secondary nucleation (*right*).

The surface plasmon resonance findings indicate robust binding between CsgC monomer and CsgA fibrils (Fig. 3). Additionally, Amylofit data fitted to CsgA ThT binding assays in the presence of varying concentrations of CsgC homologs indicate that addition of CsgC and CsgC homologs drastically affect the rate of fibril elongation (Figs. S2–S5). Taken together, it is likely that CsgC inhibits at later stages in amyloid formation through a stable interaction with fibrils, sterically preventing addition of CsgA monomers for fibril elongation or by inhibiting secondary nucleation. This mechanism is depicted in Figure 6, right side.

It is worth noting that a “secondary-nucleation dominant” model was chosen to fit the experimental data analyzed by Amylofit. Secondary nucleation is less prevalent in functional amyloids as compared to disease associated with amyloids and has not been observed in wild-type and mutant CsgA fibril formation (45, 48). Despite this, CsgA aggregation best resembles an aggregation model that accounts for both fibril growth and a form of self-replication (49). Future directions include single-molecule imaging of CsgA amyloid fibril growth to quantify individual fibril growth rates in the presence or absence of CsgC (50).

### CsgC transiently interacts with the CsgA monomer

While CsgC may inhibit CsgA fibril growth, its function *in vivo* is likely to prevent any initiation of amyloid assembly which would be cytotoxic to the cell. Therefore, we sought to determine the mechanism of CsgC inhibition of pre-nuclear CsgA. Surface plasmon resonance indicated no measurable binding between CsgC monomers and CsgA monomers (Fig. 3*B*). However, after quickly passing through a CsgC-linked column, freshly purified CsgA monomers require approximately 50 additional minutes to begin forming ThT positive species (Fig. 4, *B* and *D*). The CsgA monomers were quickly pushed through the column by hand, therefore, any interaction with immobilized CsgC was over a matter of seconds. And yet, there was a lasting impression on CsgA since aggregation was delayed (Fig. 4*D*). This suggests that a fast CsgC-CsgA monomer interaction delays CsgA monomers from adopting an aggregation-prone state.

Using IM-MS, we detected 1:1 CsgC and CsgA heterodimers, which indicate that CsgA and CsgC monomers can interact with each other (Fig. 5*B*). The heterodimer peaks were in low abundance, consistent with the concept of a transient interaction between CsgA and CsgC monomers. The heterodimer peaks are the first direct observation of an interaction between full-length CsgC and CsgA monomers.

### CsgC maintains intrinsically disordered monomeric CsgA

Much can be gleamed from focusing on the IM-MS signals associated with CsgA monomers. Multiple charge states of CsgA monomers can be seen in the mass spectrum, consistent with an intrinsically disordered protein (Fig. 5*B*). We analyzed how the CsgA monomer’s shape changed by examining the ATD over time (Fig. 5*D*, S9, *C* and *D*). The ATD profile suggests that the conformation of CsgA became more extended as aggregation progresses, a change commonly observed with other amyloid proteins as well (Fig. 5*D*, S9, *C* and *D*) (37, 38). Remarkably, the presence of CsgC in solution causes the ATD of CsgA monomers to remain consistent throughout the time course of our experiment (Fig. 5*D*, S9, *C* and *D*). Since CsgA monomers start as intrinsically disordered, CsgC must push CsgA monomers away from the aggregation-prone state by promoting or stabilizing CsgA’s intrinsically disordered fold as shown in Figure 6, left side. This is consistent with the findings from Figure 4, illustrating that transient interaction between CsgA and CsgC leads to a memorable but temporary inhibition of CsgA amyloid formation. This inhibitory mechanism likely corresponds to the functional role that CsgC plays in the periplasm during curli expression and formation. CsgC retains CsgA in an intrinsically disordered form in the periplasm to promote secretion of the soluble disordered form of CsgA, thereby preventing the formation of intracellular cytotoxic aggregates. Therefore, the transient nature of the interaction between CsgA and CsgC enables sub-stoichiometric inhibition of intracellular amyloid formation while also optimizing secretion of the functional amyloid. We propose that the inhibitory effect of CsgC on fibril elongation and/or secondary nucleation may stem from its biological role as an inhibitor of CsgA nucleation.

## Conclusion

We utilized an array of biophysical methods to show that CsgC utilizes a non-canonical chaperone mechanism by interacting transiently with its client protein and promoting an intrinsically disordered, monomeric form of CsgA to disrupt new nuclei formation in an ATP-independent fashion. This model of inhibition fits within the greater context of curli biogenesis: CsgC is a periplasmic protein tasked with inhibiting amyloid formation by CsgA within the intermembrane space to prevent the formation of cytotoxic intracellular aggregates while also allowing for efficient secretion of monomeric and intrinsically disordered CsgA for subsequent amyloid formation on the bacterial surface. We also show that CsgC is capable of inhibiting CsgA fibril formation even in the presence of CsgA nuclei, likely through stably interacting with the amyloid fibril preventing further growth. We propose that CsgC, and other similarly structured amyloid inhibitors, may represent a new class of chaperone proteins capable of potent protein remodeling activity.

## Data availability

All data generated or analyzed during this study are included in this article or are available from the corresponding author upon reasonable request.

## Supporting information

This article contains supporting information (27, 51, 52, 53, 54).

## Conflict of interest

The authors declare that they have no conflicts of interest with the contents of this article.
